# Hypercalcemia during initiation of antiretroviral therapy in human immunodeficiency virus and *Leishmania* coinfection: A case report

**DOI:** 10.1097/MD.0000000000033848

**Published:** 2023-06-16

**Authors:** Tammy Yu, Jie Tang

**Affiliations:** a Brown University, Providence, RI.

**Keywords:** human immunodeficiency virus, hypercalcemia, immune reconstitution inflammatory syndrome, leishmaniasis

## Abstract

Hypercalcemia is a common complication of many granulomatous diseases but is not typically associated with leishmaniasis. Here we report an unusual case of hypercalcemia during the initiation of antiviral therapy in a patient with acquired immunodeficiency syndrome coinfected with visceral leishmaniasis.

**Patient Concerns:**

Our patient presented with malaise and altered mental status following antiretroviral therapy initiation. He was found to have de novo hypercalcemia complicated by acute kidney injury.

**Diagnosis, interventions, and outcomes:**

An extensive workup for other etiologies of hypercalcemia was negative. The patient was ultimately thought to have hypercalcemia secondary to visceral leishmaniasis in the setting of immune reconstitution inflammatory syndrome. He was treated with intravenous volume expansion, bisphosphonates, and oral corticosteroid therapy with complete resolution.

**Lessons:**

This case highlights an unusual presentation of immune reconstitution inflammatory syndrome, in which proinflammatory cytokine signaling during the restoration of cellular immunity may have led to increased ectopic calcitriol production by granuloma macrophages, thereby altering bone-mineral metabolism and driving hypercalcemia.

## 1. Introduction

Hypercalcemia is found in 1 to 2% of the general population. When severe, it can lead to complications including arrhythmia, acute kidney injury, neurological sequelae, and death. Though primary hyperparathyroidism and malignancy account for the vast majority of cases, hypercalcemia also occurs in granulomatous diseases such as sarcoidosis, pulmonary tuberculosis, and lymphoma.^[1]^ The underlying mechanism in these diseases is excess vitamin D 1-alpha-hydroxylase activity in macrophages and giant cells found within granulomas, resulting in calcitriol overproduction.

Leishmaniasis is a complex granulomatous disease caused by protozoan parasites of the genus *Leishmania*.^[2]^ Despite this, hypercalcemia has never been reported in patients with leishmaniasis. Here, we discuss a case in which a patient with human immunodeficiency virus (HIV) and *Leishmania* coinfection developed new-onset hypercalcemia in the context of antiretroviral therapy (ART) initiation.

## 2. Case report

A 46-year-old male with a history of uncontrolled acquired immunodeficiency syndrome (AIDS) complicated by recurrent visceral leishmaniasis (VL) presented with 2 weeks of malaise, confusion, and poor appetite. Review of systems was otherwise negative, including for fever or other focal infectious symptoms. Three months ago, he had resumed taking ART with robust reduction in HIV viral load but slow recovery in CD4 count. He was on monthly amphotericin B for maintenance treatment of VL.

Exam demonstrated cachexia but was otherwise unremarkable. Laboratory workup was notable for significant acute kidney injury with creatinine 4.34 mg/dL (baseline 0.9), corrected calcium of 13.7 mg/dL (previously 9–10), and pancytopenia. The patient underwent a broad hypercalcemia workup. Parathyroid hormone was appropriately suppressed (<6, normal 18–80 pg/mL), and calcidiol was low (8.5, normal 30.0–100.0 ng/mL), ruling out primary hyperparathyroidism and dietary vitamin D toxicosis. His calcitriol, however, was inappropriately normal (40, normal 18–72 pg/mL), and his angiotensin-converting enzyme level was elevated (221, normal 9–67 units/L), in keeping with granulomatous disease. Serum protein immunofixation revealed a monoclonal IgG-lambda protein, although the kappa/lambda ratio was only trivially elevated (1.93, normal 0.26–1.65).

He ultimately underwent a repeat bone marrow biopsy that demonstrated non-necrotizing granulomas. The plasma cell population was polyclonal, ruling out multiple myeloma. His marrow was positive for *Leishmania donovani*/*infantum* by polymerase chain reaction. Workup for other infections, including Histoplasma and Aspergillus antigen as well as acid-fast bacillus and fungal cultures, was negative.

During his month-long admission, his CD4 count rebounded from 18 to 354. Given the rapid rise in CD4 count after ART initiation, as well as persistent bone marrow VL infection despite recent treatment, he was diagnosed with immune reconstitution inflammatory syndrome (IRIS). As his workup had ruled out other etiologies of hypercalcemia, he was ultimately thought to have developed granuloma-associated hypercalcemia in the setting of VL-triggered IRIS. Hypercalcemia and acute renal failure likely contributed to his presenting symptoms of fatigue and malaise.

**Figure 1.**
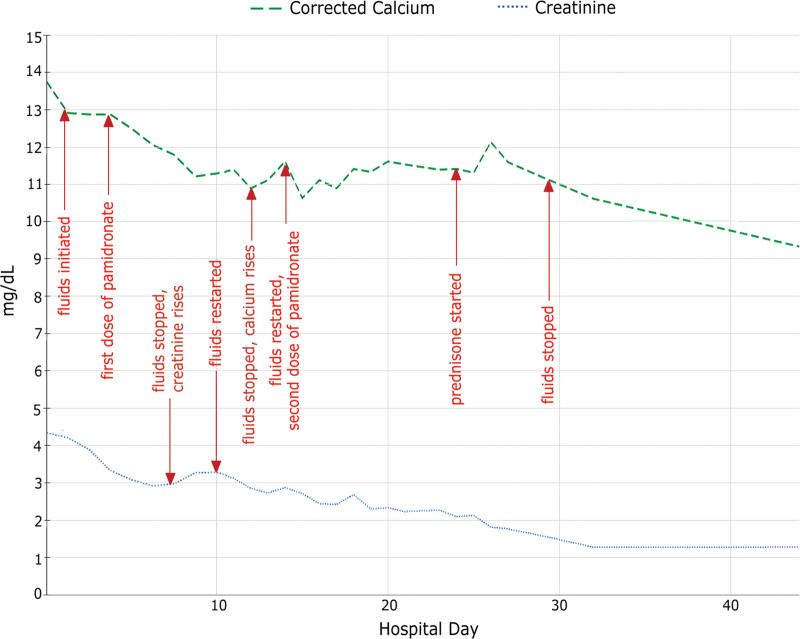
Creatinine and serum calcium trend over treatment course. Arrows denote the timing of interventions.

Treatment was initiated with aggressive volume expansion and pamidronate, leading to improvement in both hypercalcemia and renal clearance. However, despite improvements in appetite and PO intake, he remained dependent on intravenous fluids over the next several weeks, as attempts at weaning or stopping continuous fluids led to rises in his calcium and creatinine levels. Prednisone was subsequently started for treatment of both IRIS and granulomatous disease. This led to a sustained normalization of his calcium levels and allowed him to wean off fluids (Fig. 1). At discharge, he was continued on a long prednisone taper with amphotericin B as secondary prophylaxis for VL.

## 3. Discussion

Leishmaniasis is a protozoan infection caused by various species in the genus *Leishmania*. Clinical manifestations of leishmaniasis range from asymptomatic infection to a spectrum of overt disease, including both cutaneous infection and disseminated visceral disease.^[3,4]^ Despite aggressive vector control and better access to treatment, leishmaniasis remains a global health threat, with an estimated annual incidence of 0.7 to 1 million cases.^[3,5]^ Coinfection with HIV in endemic countries is also an increasing concern.^[6]^

Granulomas are a classic pathologic feature of leishmaniasis,^[2]^ representing organized collections of immune cells responding to chronic antigenic stimulation. While they allow the host immune system to contain the pathogen and prevent disease dissemination, they can also become reservoirs of pathogen persistence, allowing for the spread of pathogens into the bone marrow, lymph nodes, liver, and spleen when immunity is compromised.^[6]^

Macrophages initiate granuloma formation and are a key component of mature granulomas. In response to cytokine signaling, they can adopt either a proinflammatory M1 phenotype or a pro-tissue repair M2 phenotype. Whereas M1 macrophages promote more effective pathogen killing, excess proinflammatory signaling can lead to tissue damage. Conversely, M2 macrophage stimulation mediates immune tolerance and repair, but can also impair pathogen eradication. Granulomas can be dominated by either M1 or M2 macrophages. The balance between pro- and anti-inflammatory immune responses at the site of granulomatous infection is crucial for controlling infection while limiting collateral tissue damage.^[7]^

Parasitic infections generally induce strong type 2 immunity and production of type 2 cytokines, leading to an M2-predominant response in granulomas.^[8]^ In an animal model of VL, the M2 phenotype was predominant in macrophages taken from granulomas and inflammatory infiltrates in skin, lymph nodes, and spleens. The highest proportions of M2 macrophages coincided with the highest parasite loads.^[9]^

Hypercalcemia is a common finding in many granulomatous disorders, both infectious and noninfectious.^[10–12]^ Interestingly, leishmaniasis is not among these. A recent study has demonstrated that proinflammatory M1 macrophages express significantly higher levels of vitamin D 1-alpha-hydroxylase than activated M2 counterparts, indicating that M1 macrophages might have a higher capacity to convert circulating calcidiol to its active form, calcitriol.^[13]^ This could explain why hypercalcemia is not usually seen in leishmaniasis, where M2 macrophages predominate. Indeed, during his initial presentation with VL several months prior to this admission, our patient had normal serum calcium levels. However, hypercalcemia developed after ART was started for the treatment of HIV/AIDS, potentially triggered by IRIS.

IRIS is a well-recognized phenomenon in patients infected with HIV. ART that restores CD4 T-cell responses can have the paradoxical effect of unmasking or reactivating occult infections.^[7]^ Low CD4 + cell counts, high infection burden, and a short interval between the treatment of opportunistic infection and the start of ART are risk factors for IRIS.^[14]^ Proinflammatory cytokines and chemokines are important contributors to the exaggerated immune response in IRIS.^[15,16]^ These cytokines boost proinflammatory T-helper type 1 and M1 macrophage responses, and thus also theoretically enhance 1-alpha-hydroxylase expression.^[14,15]^ Our patient’s new-onset hypercalcemia after ART initiation suggests that immune reconstitution may have altered cytokine signaling, thus leading to excess production of active vitamin D.

Corticosteroids are the cornerstone of treatment for granulomatous disorder-associated hypercalcemia. Prednisone 20 to 40 mg daily can be started to reduce the production of endogenous calcitriol, with circulating levels typically declining within the first 3 to 5 days. Once hypercalcemia is controlled, prednisone can be tapered over a period of 4 to 6 weeks. In cases of treatment failure or steroid intolerance, chloroquine or hydroxychloroquine can be used.^[10]^ Corticosteroids, along with targeted antimicrobial therapy, can also be helpful in the symptomatic management of IRIS.^[14]^ ART should not be interrupted except in cases of severe or life-threatening disease.^[17]^ Even after successful control of the initial infection, parasites can remain dormant during the latent phase, providing a source for subsequent reactivation of disease in immunocompromised patients. Therefore, lifelong monitoring and preventive therapies may be indicated.

Although hypercalcemia has not previously been reported in leishmaniasis, our case suggests that this may be a potential complication of IRIS when ART is initiated in patients with HIV and granuloma-forming coinfections, driven by excess type 1 immune signaling in the setting of immune reconstitution after ART initiation. Our patient’s workup for other etiologies of hypercalcemia, including exogenous vitamin D ingestion, primary hyperparathyroidism, multiple myeloma, tuberculosis, and sarcoidosis, was otherwise negative. His response to prednisone treatment also suggests that his hypercalcemia was driven by a steroid-responsive condition such as granuloma macrophage activation. Nevertheless, this case represents only a single instance of hypercalcemia in VL-associated IRIS, and further observational studies are needed to establish a definitive correlation between the 2.

We report this novel case of hypercalcemia in hopes of expanding the literature on the various potential manifestations of VL, particularly in the setting of IRIS and AIDS. Increased awareness of and prompt treatment of hypercalcemia is needed to optimize outcomes in this patient population.

## Author contributions

**Conceptualization:** Jie Tang.

**Writing – original draft:** Tammy Yu, Jie Tang.

**Writing – review & editing:** Tammy Yu.
